# Convergent Evolution Dynamics of SARS-CoV-2 and HIV Surface Envelope Glycoproteins Driven by Host Cell Surface Receptors and Lipid Rafts: Lessons for the Future

**DOI:** 10.3390/ijms24031923

**Published:** 2023-01-18

**Authors:** Jacques Fantini, Henri Chahinian, Nouara Yahi

**Affiliations:** INSERM UMR_S 1072, Aix Marseille University, 13015 Marseille, France

**Keywords:** virus evolution, HIV-1, SARS-CoV-2, electrostatic surface potential, ganglioside, lipid raft, quasi-species, receptor

## Abstract

Although very different, in terms of their genomic organization, their enzymatic proteins, and their structural proteins, HIV and SARS-CoV-2 have an extraordinary evolutionary potential in common. Faced with various selection pressures that may be generated by treatments or immune responses, these RNA viruses demonstrate very high adaptive capacities, which result in the continuous emergence of variants and quasi-species. In this retrospective analysis of viral proteins, ensuring the adhesion of these viruses to the plasma membrane of host cells, we highlight many common points that suggest the convergent mechanisms of evolution. HIV and SARS-CoV-2 first recognize a lipid raft microdomain that acts as a landing strip for viral particles on the host cell surface. In the case of mucosal cells, which are the primary targets of both viruses, these microdomains are enriched in anionic glycolipids (gangliosides) forming a global electronegative field. Both viruses use lipid rafts to surf on the cell surface in search of a protein receptor able to trigger the fusion process. This implies that viral envelope proteins are both geometrically and electrically compatible to the biomolecules they select to invade host cells. In the present study, we identify the surface electrostatic potential as a critical parameter controlling the convergent evolution dynamics of HIV-1 and SARS-CoV-2 surface envelope proteins, and we discuss the impact of this parameter on the phenotypic properties of both viruses. The virological data accumulated since the emergence of HIV in the early 1980s should help us to face present and future virus pandemics.

## 1. Introduction

Virus-cell interactions during the early stages of the infection cycle of enveloped viruses have been the subject of numerous studies for several decades. The COVID-19 pandemic has mobilized the efforts of the scientific community around the world. Among these researchers, two of us (N.Y. and J.F.) extensively studied the molecular mechanisms associated with the infection of human cells by HIV-1 and other retroviruses. Nouara Yahi began her work on HIV by joining, in 1988, the retrovirus laboratory newly created in Marseille by Jean-Claude Chermann, one of the co-discoverers of the AIDS virus at the Pasteur Institute in Paris. She was in charge of HIV strain isolation [1] and antiviral drug testing [2]. Jacques Fantini joined the team in 1990. The two then started to study the infection of human epithelial intestinal cells by HIV-1 [3], a project which would eventually solve two issues: (i) the identification of galactosylceramide (GalCer) as an alternative HIV-1 receptor in these CD4-negative cells [4], shortly after the group of F. Gonzalez-Scarano in Philadelphia identified it as the portal of entry of HIV-1 in neural cells [5]; (ii) a virotoxin-induced signal transduction pathway accounting for the puzzling HIV-associated enteropathy: HIV-1 surface envelope glycoprotein gp120 binding GalCer, intracellular Ca^2+^ release, microtubule disruption, and impaired absorption functions [6,7,8,9,10]. Subsequently, our team took part in research that led to the identification of HIV-1 fusion co-receptors by identifying the first glycolipid cofactors Gb3 [11,12] and GM3 [13,14] necessary for the infection of lymphocytes and macrophages [15]. In 1997, K. Simons and E. Ikonen proposed the lipid raft concept, which modified our vision of the plasma membrane [16]. Instead of considering glycolipids as individual entities, we have integrated the fact that glycolipids and cholesterol are concentrated in the microdomains that float as rafts in the more liquid bulk membrane [17]. In light of this new paradigm, we proposed that the HIV-1 particle first binds to its CD4 receptor, associated with a lipid raft, then moves like a surf on the plasma membrane until reaching a functional coreceptor [11,18,19], chiefly CCR5 [20] or CXCR4 [21]. In full agreement with this concept, lipid raft disruption by pharmacological agents and inhibitors of glycosphingolipid synthesis could prevent HIV-1 infection, without targeting any specific HIV-1 receptor or co-receptor [22,23,24].

In 2002, we highlighted a striking analogy between HIV-1 gp120 and the amyloid proteins implicated in neurodegenerative diseases (Alzheimer’s, Parkinson’s, Creutzfeldt–Jakob). All these proteins can bind to raft glycolipids, and we have discovered the domain responsible for these interactions [25]. We named this domain the sphingolipid binding domain (SBD), given that rafts are made up of sphingolipids and cholesterol [17]. In the case of amyloid proteins, the sphingolipids involved in the interaction with the plasma membrane of brain cells are gangliosides, and the domain, thus, became the ganglioside binding domain (GBD) [26].

When SARS-CoV-2 emerged in 2019, we got back into virology to contribute to research efforts on this new virus [27]. In the first half of 2020, the confinements have been particularly conducive to in silico studies focusing on different aspects of SARS-CoV-2 infection, but more particularly, the analysis of spike protein. At that time, there was still no vaccine, and there was a major interest in repositioned drugs that could block the replication of this virus. Among these potential antivirals, chloroquine and its derivative hydroxychloroquine focused the attention of many researchers, based on encouraging in vitro results [28]. We then decided to start in silico research on the possible mechanisms of the antiviral effects of these compounds. Since we did not find hydroxychloroquine binding sites on the spike protein of SARS-CoV-2, we wondered if this antiviral could bind to lipid rafts and competitively block the virus from binding to the host cell surface. This is how we discovered a new mechanism of action of hydroxychloroquine, which recognizes the GM1 gangliosides expressed by respiratory epithelial cells and competitively blocks SARS-CoV-2 binding to these cells [29]. In the same publication, we showed that the N-terminal domain (NTD) of the spike protein has a large flat contact area for lipid rafts. Then, we showed that azithromycin binds to this flat NTD surface, suggesting interesting synergistic properties of hydroxychloroquine and azithromycin as an antiviral combination [30]. Indeed, this association works in vitro [28], confirming the accuracy of our in silico analyses.

In the fall of 2020, the first variants of SARS-CoV-2 emerged [31,32], perplexing many specialists counting on the higher stability of coronavirus genomes, compared to other RNA viruses [33]. We then undertook a structural cracking of the spike proteins of the variants of concern as they emerged in the world. This comparative analysis led to a new concept, not taking the affinity of the virus for its ACE2 receptor into account, but the kinetic parameters based on the surface electrostatic potential of the regions of the spike protein facing the plasma membrane of the host cell [34].

SARS-CoV-2 and HIV-1 are both rapidly evolving RNA viruses that mutate within hosts and exist as viral quasi-species (defined as a set of related variants occurring in the same infected individual) [35,36,37,38]. Quasi-species usually refer to intra-host viral diversity, whereas variants reflect inter-host diversity. In this review, we first propose a survey of the data obtained on SARS-CoV-2 variants (from the original Wuhan strain to Omicron) [39]. Then, we will do a similar work with representative HIV-1 strains to determine if there are elements of convergence that can help us face the possible emergence of new viruses. To our surprise, this thematic has not been covered in depth, with 391 results of the Pubmed query “HIV-1 SARS-CoV-2” (https://pubmed.ncbi.nlm.nih.gov/, accessed on 19 November 2022). Very few studies compared the properties of both viruses [40,41,42,43,44,45,46,47]. Moreover, a recent review focused on the mechanisms of SARS-CoV-2 entry into cells [48] mentioned neither gangliosides nor lipid rafts. This explains why we decided to carry out this comparative analysis emphasizing the role of lipid rafts, in light of our previous work on HIV, combined with our recent contribution to the SARS-CoV-2 research field.

## 2. Structural and Functional Analysis of the SARS-CoV-2 Spike Protein

**Figure 1:**
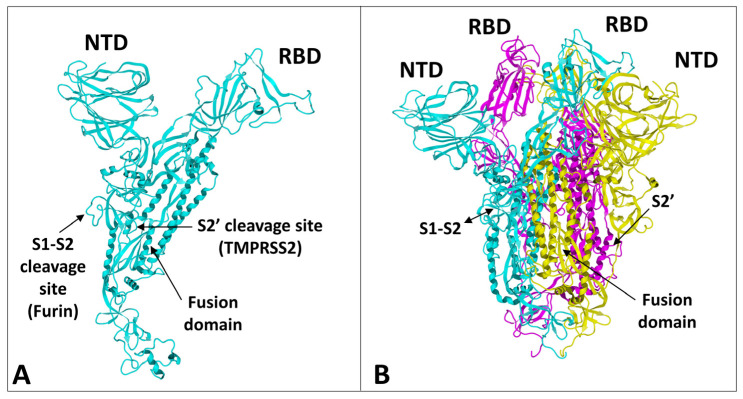
Structural features of SARS-CoV-2 spike protein monomer (**A**) and pre-fusion trimer (**B**). NTD, N-terminal domain; RBD, receptor binding domain. In the trimer, the subunits ribbons are colored in cyan, yellow, and magenta. The two proteolytic cleavage sites (S1–S2 and S2′) are indicated in the monomer and (when visible) in the trimer. The models were modified from pdb file 7 bnm.

**Figure 2:**
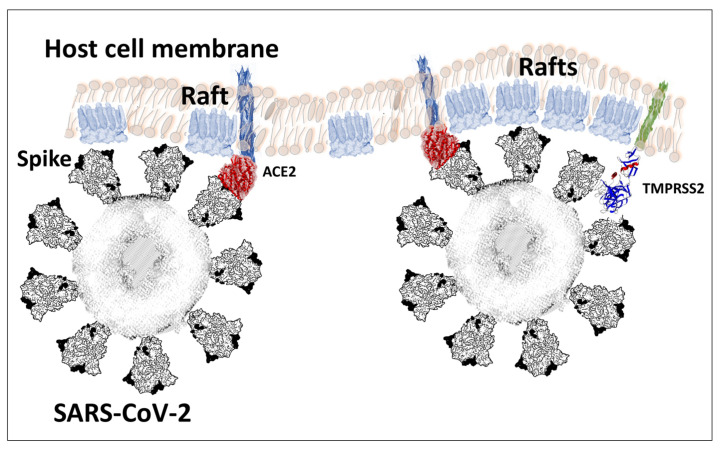
Spacecraft model of SARS-CoV-2 docking to the host cell membrane. The first step is the attachment of a spike trimer to lipid rafts enriched in ganglioside GM1. The N-terminal domain (NTD) of the spike protein controls GM1 recognition. The second step is the coordinated movement of the raft-virus complex, until it reaches the ACE2 receptor. A conformational change of the spike protein is required to unmask the RBD for ACE2 binding. The raft-virus-ACE2 complex then moves to reach the cellular protease TMPRSS2. Lipid rafts assist this thermodynamically controlled mechanism at all steps.

The spike protein of SARS-CoV-2 is a large glycoprotein synthesized as a precursor containing 1273 amino acid residues for the original Wuhan strain (https://www.uniprot.org/uniprotkb/P0DTC2/entry#sequences accessed on 15 November 2022). The first 18 amino acids form the signal sequence, which is cleaved to generate the mature form encompassing residues 19-1273. As shown in Figure 1A, the protein has a typical Y shape, whose upper branches correspond to the N-terminal domain (NTD) and the receptor-binding domain (RBD). The lower trunk of the Y displays two proteolytic sites: (i) S1–S2, which is cleaved by furin in the Golgi apparatus during biosynthesis and maturation in the infected cell, and (ii) S2′, an additional cleavage site that is essential for the virus to fuse at the plasma membrane of human lung cells [48,49]. The cleavage of S1–S2 by furin generates two subunits (S1 and S2) that remain non covalently attached. The S2′ site is cleaved by type II transmembrane serine protease 2 (TMPRSS2) at the cell surface or by cathepsins in the endosome [44,48]. In the absence of the furin site, or if the mutations render this site non-functional [50], the alternative for the virus is, thus, to gain entry into the cell by endocytosis, according to a classic mechanism shared by many coronaviruses [48,51]. The S1 subunit contains the NTD and the RBD, which bind to the cell surface, whereas the S2 subunit possesses the machinery necessary for the fusion between the virus envelope and the plasma membrane of the host cell [52]. The RBDs and the fusion machinery cluster in the center of the trimer, while the NTDs are pushed to the sides. There are two forms of the trimeric spike, the closed form and the open form [52]. The closed form (Figure 1B) must undergo a conformational change to make the central RBDs accessible and, thus, allow them to interact with a cellular receptor, primarily ACE2 [53]. Therefore, the attachment of the spike protein to the ACE2 receptor cannot be the first step in the process of adhesion of the virus to the surface of the host cell [27]. It is obvious that it is, indeed, the closed form of the spike that must first attach itself to the cell, which will trigger the conformational change, which will subsequently allow attachment to ACE2. In our previous articles, we compared this mechanism to docking a spacecraft on a space station [27]. First, each NTD domain seeks a favorable landing area that we have identified as a lipid raft, i.e., a plasma membrane microdomain enriched in gangliosides and cholesterol [17] (Figure 2). Indeed, we have shown that NTD has an excellent affinity for GM1 gangliosides, including the acetylated GM1 derivatives, which are particularly abundant on the surface of respiratory mucosal cells [34]. Since there are 3 NTDs per spike trimer, this multiplies the chances of adhesion to a lipid raft, which ensures the initial attachment of the virus to the plasma membrane of the target cell [54]. The first trimer sticks to a raft, causing a local deformation of the membrane, which invaginates (Figure 2). This allows other virus trimers to interact with vicinal rafts. The virus will then sail on these rafts until it encounters the ACE2 receptor, which is, itself, a raft-associated protein [55]. This fast “surfing” process [56] facilitates the formation of a trimolecular complex consisting of ACE2, raft gangliosides, and the spike protein [29]. The next step is a conformational change of the spike trimer, which triggers the unmasking of the RBD [57]. Finally, the spike protein is cleaved at the S2′ site by TMPRSS2 [58], which in turn initiates the fusion process controlled by the S2 subunit [59]. We are, thus, dealing with a perfectly controlled thermodynamic mechanism, which leads inexorably from adhesion to fusion. The selection pressure that controls the evolution of SARS-CoV-2 variants at the level of the spike protein will, therefore, apply to each of these steps by independently targeting the parts of the spike protein involved. However, the parameters on which these selection pressures will act will not be the same, depending on the targeted domain. In the case of the NTD, which controls the initial step of adhesion of the virus to a lipid raft, the selection pressure will be exerted preferentially on the kinetics of adhesion. For the RBD, there will be a combination of direct effects and indirect effects. The direct effects will concern the kinetics of the RBD-ACE2 interaction, but also the affinity parameter [34]. The indirect effects will be caused by mutations facilitating conformational unmasking of the RBD, such as the D614G mutation [39]. However, the other mutations may, on the contrary, stabilize the trimer in its closed conformation, thus increasing the resistance of the virus to extreme conditions and favoring routes of contamination other than via the respiratory mucosa (for example by ingestion) [60]. The furin site may be modulated by mutations facilitating proteolytic cleavage, or instead, minimizing it, as we will see in the case of the Omicron variants. Finally, mutations in the helix domains involved in the fusion mechanism may also affect the infectivity of the virus. Yet, the analysis of SARS-CoV-2 mutations is further complicated by two parameters that must also be taken into account. First, each mutation cannot be analyzed independently of other mutations in the same functional domain [60]. Thus, the impact of a single mutation will depend not only on its own effect on the structure of the protein, but also on its contribution to a global mutational pattern. Secondly, it must be considered that, in many countries, the anti-COVID-19 vaccination rate is very high, which means that the vaccine must also be considered as a selection pressure [61]. The recent finding of a higher intra-host diversity among vaccinated individuals is also in favor of a potential vaccine-induced immune pressure [35]. Thus, many mutations are selected not on the basis of the advantage they confer, in terms of virus infectivity, but rather for their contribution to immune escape. People infected several times by the virus are also affected by this phenomenon, whether they are vaccinated or not. In summary, we face a system of two equations (mutational profile and immune escape) with four unknowns (NTD, RBD, cleavage site, and fusion machinery). The problem being posed, let us now see what the analysis of these different parameters has taught us about the successive waves of SARS-CoV-2 variants.

## 3. Structural Dynamics of SARS-CoV-2 Spike Protein Evolution

**Figure 3:**
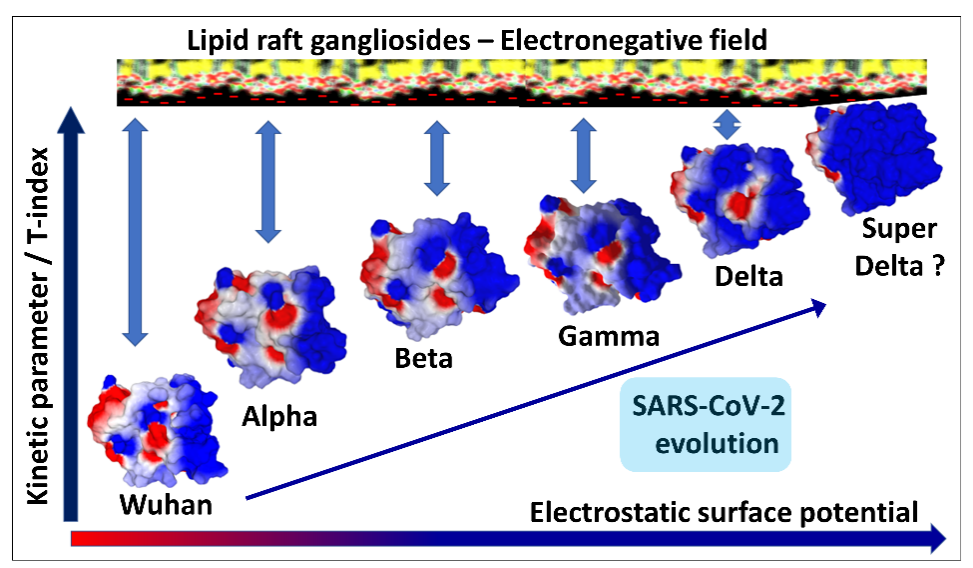
Electrostatic surface potential evolution in the NTD and RBD of SARS-CoV-2 variants (from the original Wuhan strain to Delta). The successive variants of SARS-CoV-2 show increased electrostatic potential of the N-terminal domain (NTD) surface and of the transmissibility T-index. This allows a faster attachment to the electronegatively charged surface of lipid rafts, due to the presence of gangliosides. However, Delta has such a high electrostatic potential that it cannot be further increased. Thus, Delta was a dead-end evolution. Indeed, a “super Delta” variant did not appear after Delta. Blue, electropositive; red, electronegative; white, neutral.

**Figure 4:**
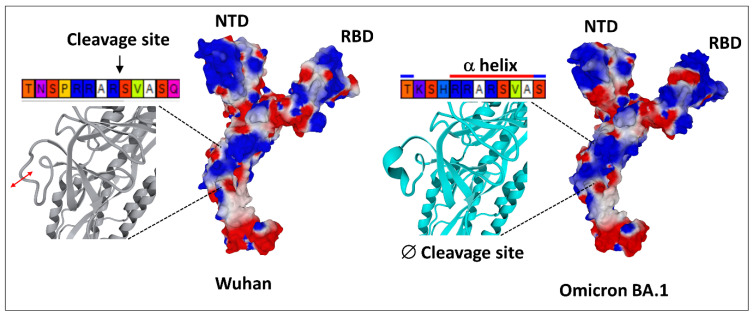
The singularity of Omicron SARS-CoV-2 variant. In the Wuhan strain (left panel), the S1–S2 cleavage site TNSPRRAR*SVASQ is flexible and functional. In the first Omicron variant (BA.1, right panel), the S1–S2 cleavage site is structured and, thus, less flexible and non-functional. One can also note the curved surface of the N-terminal domain (NTD) of Omicron BA.1, compared with the flatter surface of this domain in the Wuhan spike. RBD, receptor binding domain. The structures were modeled from pdb file 7 bnm.

Schematically, the NTD has two fundamental properties that explain why this domain is particularly efficient in targeting the gangliosides of the lipid rafts: (i) its contact surface with the cell is flat [29,56], and (ii) it is globally electropositive [34]. This combination of geometrical and electrostatic parameters is truly the winning formula for binding to lipid rafts, which are well-demarcated flat and electronegative landing zones [62]. Each GM1 ganglioside has an ionized sialic acid and, therefore, has a negative charge at physiological pH. The repetition of this negative charge over the raft gives these membrane domains a global negative electrostatic potential [62]. Under these conditions, the more the NTD is electropositive, the faster it will interact with the raft. Mutations that increase the electropositivity of the NTD, therefore, directly affect the kinetics of interaction of the virus with the raft [34]. In the order of appearance of SARS-CoV-2 variants, from the original Wuhan strain to the Delta variant, the electrostatic potential underwent a steady increase, becoming more and more electropositive (Figure 3) [34]. However, the electrostatic potential cannot increase indefinitely because, beyond a certain value, the virus could stick too strongly to the membrane, which would have the effect of making it non-infectious (Figure 3). Therefore, it was clear that the Delta variant was the final outcome and no virus could supplant it, except to go back and compensate for the decrease in surface potential by another parameter. This is how the first Omicron variant appeared, when Delta was still predominant [63]. It remained so in several countries, especially those where vaccination was the least widespread, such as South Africa [64]. We can clearly see that the analysis of variants is multiparametric and that it is sometimes necessary to look elsewhere, other than in the virus itself, to find the reasons explaining the emergence of one variant compared to another. Thus, it is in the most vaccinated countries, such as France or Denmark, that the wave of Omicron was the strongest, even though Delta was still very present [65,66,67]. It is, therefore, most likely due to an immune escape phenomenon that the first Omicron variant was able to succeed Delta, with the neutralizing antibodies induced by the vaccine being notably less effective against Omicron, compared to Delta [68,69]. A very strong argument in favor of this interpretation is given by the in vitro infection kinetics comparing the Delta and Omicron in Calu-3 cells, which have high level expressions of TMPRSS2 and in (TMPRSS2)-overexpressing VeroE6 cells [70]. In a competition assay with these cells, Delta outcompeted Omicron [70]. This means that, on a strictly virological level, Omicron has no decisive advantage over Delta. For Omicron to take over Delta, it needed outside help, the natural or vaccinal immune response. However, as we mentioned above, the selection pressures are multiple and Omicron has acquired a faculty that Delta did not have, nor the other variants that preceded it: an inoperative furin site (S1–S2) condemning the virus to enter the cells through endocytosis [70,71]. This resistance to furin cleavage is manifested at the level of the cleavage zone by a structuring that reduces the flexibility of the substrate loop (Figure 4).

**Figure 5:**
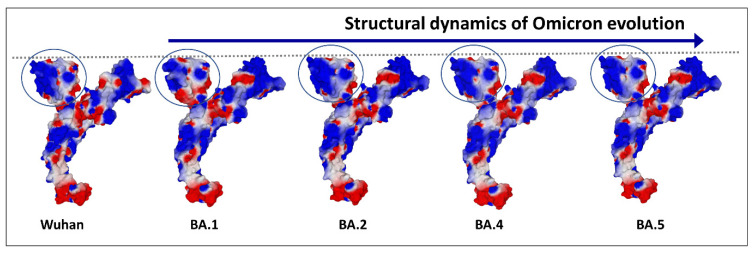
Evolution of the electrostatic surface potential in the Omicron lineage. The N-terminal domain (NTD) is indicated by a circle. Note that the curved surface of BA.1 has evolved to a flatter one from BA.2. Apart from the NTD and the RBD, the electrostatic surface potential of the spike protein does not display significant changes from Wuhan to Omicron BA.5.

Finally, Omicron’s mutational program is really a jigsaw puzzle, with mutations that seem to go all over the place [72]. The electropositive surface potential of the NTD is diminished, compared to Delta, but that of the RBD is increased. The contact surface of the NTD is slightly rounded (Figure 4), but this problem will be solved by successive Omicron variants, from BA.2 to BA.5 (Figure 5) and BQ.1.1 today.

In order to provide a comparative analysis of SARS-CoV-2 variants, we have proposed a transmissibility index (T-index) taking into account the kinetic parameters (surface electrostatic potential of NTD and RBD) and affinity (NTD-gangliosides and RBD-ACE2 interactions) [34,72,73]. This index clearly accounts for the evolution of SARS-CoV-2 from the original Wuhan strain to the Delta variant, with each new variant having a higher T-index than its predecessor (Figure 3). The arrival of Omicron has changed the situation, since this new line of variants has a lower T-index than Delta, with its success being due both to its mechanism of entry by endocytosis (mutations of the furin site) and to the immune escape.

One of the most intriguing aspects of the structural dynamics of the evolution of SARS-CoV-2 variants is the relative rarity of insertions and deletions, which are, nevertheless, the hallmark of RNA viruses, including HIV [74,75,76]. In fact, these events also exist for SARS-CoV-2, but they are concentrated in hot spots and, in particular, at the level of the NTD [74] and, more precisely, at the level of the contact surface with the rafts [34]. One can wonder about the significance of these rearrangements in these zones of interaction with lipid rafts. If we consider the selection pressure induced by the neutralizing antibodies recognizing these areas, the immune escape requires mutations decreasing the affinity of these antibodies for the NTD [73]. However, ultimately, these neutralizing antibodies are kind of mirrors of the NTD’s contact surface: geometrically flat and electrostatically negatively charged, similar to rafts [27]. Under these conditions, any modification of the epitopes recognized by these antibodies could also cause a decrease of the affinity of the NTD for the rafts and, therefore, a disadvantage for the virus. The deletions in the NTD can then be interpreted as a loss of epitope, without major consequences for the binding to the rafts. The virus becomes resistant to neutralizing antibodies, while remaining infectious.

Despite the complexity of the selection pressures that determine the emergence of SARS-CoV-2 variants, the mechanisms affected by the mutations of the spike protein remain perfectly explainable. Among these mechanisms, the surface electrostatic potential plays a major role, which is, in fact, shared by many viruses, such as HIV, which we are going to study now.

## 4. Structural and Functional Analysis of HIV-1 gp120 Surface Envelope Glycoprotein

**Figure 6:**
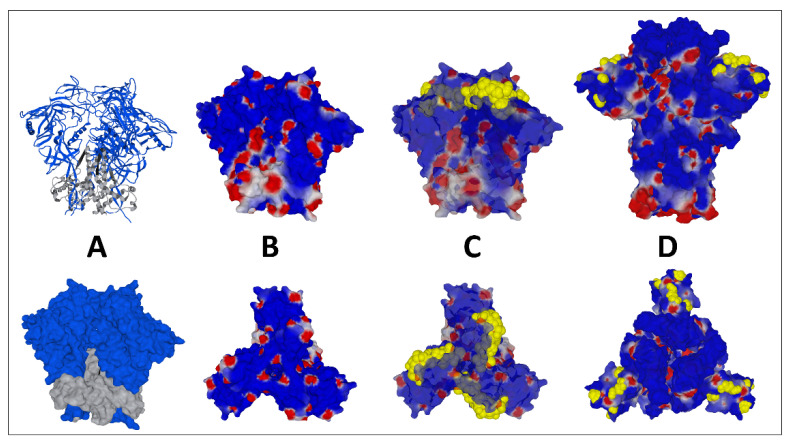
Structural features of HIV-1 gp120 surface envelope glycoprotein and gp120-gp41 trimers, compared with SARS-CoV-2. (**A**) Ribbon structure (upper panel) and surface representation of the trimeric HIV-1 gp41-gp120 complex (pdb file 6nqd). (**B**) Electrostatic surface potential of the trimeric HIV-1 gp41-gp120 complex (side view in the upper panel, top view in the lower panel). (**C**) Same as in (**B**), with V3 loop amino acid residues represented in yellow atomic spheres. (**D**). SARS-CoV-2 Omicron BA.1 trimeric spike at the same scale (modeled from pdf file 7 bnm). Amino acid residues of the N-terminal domain (NTD) that are critical for binding to raft gangliosides are represented in yellow atomic spheres.

**Figure 7:**
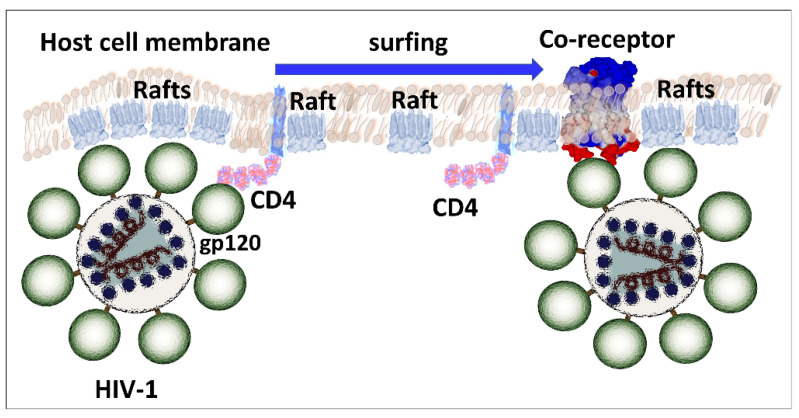
Surfing model HIV-1 binding to the plasma membrane of host cells. After initial attachment to lipid rafts through glycosphingolipid-V3 loop interactions, gp120 interacts with the CD4 receptor in a lipid raft environment. The trimolecular complex (raft-virus-CD4) then sails until finding a raft displaying an appropriate coreceptor (CCR5 or CXCR4). At this stage, CD4 detaches from the complex, allowing the V3 loop to bind to the coreceptor, which is flush to the membrane.

**Figure 8:**
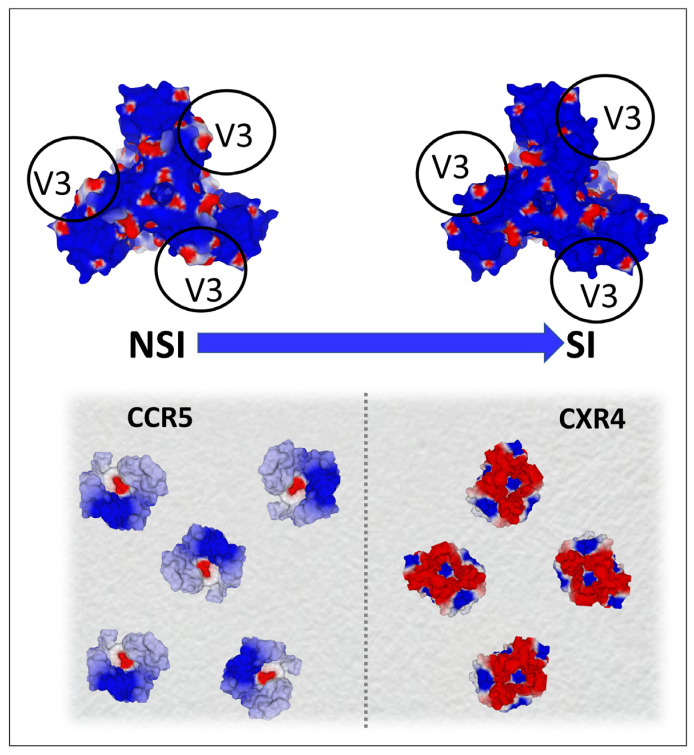
Electrostatic surface potential of CCR5 (pdb file 7f1t) and CXCR4 (pdb file 3oe0) co-receptors as an evolutionary driving force for HIV-1 evolution. The shift from non-syncytium-inducing (NSI) to syncytium-inducing (SI) strains of HIV-1 is associated with a coreceptor switch (from CCR5 to CXCR4). SI strains are more electropositive than NSI strains, due to the accumulation of mutations which increase the net charge of the V3 loop of gp120 (upper panel). This net positive charge increase renders the virus compatible with CXCR4, which is far more electronegative than CCR5 (as shown in the lower panel representing the surface of a lymphocyte). Blue, electropositive; red, electronegative; white, neutral.

In the case of HIV-1, the molecular details of the mechanisms of entry are different, but the strategy is globally similar to the one used by SARS-CoV-2. The surface envelope glycoproteins that constitute the trimeric spike of HIV-1 [77] are already cleaved from a unique precursor, gp160 [78]. This precursor is cleaved by a cellular protease before the assembly of the viral particle at the plasma membrane of the infected cells [79]. Two glycoproteins are generated (Figure 6), the surface envelope g120 (SU), which is equivalent to S1 for SARS-CoV-2, and the transmembrane gp41 (TM), which is equivalent to S2 [80]. The amino acid residues that are critical for the binding to raft gangliosides (V3 loop for HIV-1 and part of the NTD for SARS-CoV-2) are highlighted in yellow. Although the binding to lipid rafts is typically an induced fit mechanism [81], a significant part of the amino acid chain is exposed on the surface of the viral glycoproteins, allowing for fast contact with raft gangliosides. The sequence of events leading to HIV-1 entry is, however, very similar to the fusion process of SARS-CoV-2 (Figure 7). We elucidated and published this mechanism as early as 1998 [19], before it was confirmed by many subsequent studies [14,82,83,84,85,86,87,88]. The HIV-1 virion first binds to a raft, via its gp120 V3 loop domain, which, although not totally exposed on the surface of the viral glycoprotein, is sufficiently accessible to engage functional virus-raft contacts. Since the CD4 receptor is also located in a raft [89,90], this very first step facilitates gp120-CD4 binding. A conformational change then totally unmasks the V3 loop of gp120 [91,92]. The V3 loop keeps the virus firmly attached to the raft through direct interactions with accessory glycosphingolipids (Gb3 and/or GM3) [11,13,15,19]. The virus will then sail on the cell surface (surfing step) [11,15,87] until it encounters a co-receptor, CCR5 or CXCR4, also recognized by the V3 loop [93]. The V3-coreceptor interaction disconnects CD4 from the complex and triggers the last conformational change of gp41, which eventually activates the fusion machinery [94]. As in the case of SARS-CoV-2, infection can only occur if the rafts are fully functional and free to move on the cell surface. An important specificity of HIV-1 concerns the choice of the coreceptor, CCR5 or CXCR4. Initially, HIV-1 were classified according to a phenotypic criterion and the ability to induce syncytia in cultures of infected cells [95]. The syncytium-inducing (SI) viruses appear in infected patients after the non-syncytium-inducing (NSI) viruses from which they derive by accumulation of mutations at the level of the V3 loop [96]. The more aggressive a virus is, the more syncytia it forms and the more mutations it presents in its V3 loop [97]. In general, NSI viruses recognize CCR5 [98] and SI viruses recognize CXCR4 [99]. Interestingly, this coreceptor switch is strongly correlated with an increase in the surface electrostatic potential of the V3 domain [100,101]. This increase is consistent with the fact that the surface potential of CXCR4 is much more electronegative than that of CCR5 (Figure 8) [102].

## 5. Structural Dynamics of HIV-1 gp120 Surface Envelope Glycoprotein Evolution

It, therefore, appears that the evolution of SARS-CoV-2 and HIV-1 variants and quasi-species is driven by an adaptation of the contact surfaces of the virus with the areas and membrane receptors controlling the adhesion of viral particles to target cells. In fact, the analysis of the evolution of the V3 loop of HIV-1 gp120 shows an accumulation of basic amino acid residues (lysine and arginine), which increase the electropositivity of this domain [103]. It is this evolution of the surface potential that allows the virus to sequentially use the CCCR5 then CXCR4 co-receptors, while maintaining a good attractiveness for lipid rafts. From this point of view, the virus behaves like an evolutionary probe, capable of estimating the electrostatic potential of its targets and of making its own potential evolve, according to this parameter. Interestingly, this important feature was established several years before HIV-1 coreceptors were identified [104]. Indeed, the evolution of the V3 loop sequences shows a progressive enrichment in basic amino acids. As a result, a strong correlation can be established between the net charge of the V3 loop and the type of coreceptor used. Below a net charge of +3, CCR5 will be preferred. Above the value +4, the virus will then be able to use CXCR4 [100]. Viruses able to use both CCR5 and CXCR4 (referred to as R5X 4 strains) have an intermediary net charge in the 3–4 range [100].

At the phenotypic level, quasi-species using CXCR4 are more aggressive, and they can infect cellular targets, other than T4 lymphocytes and macrophages. However, since secreted gp120 has virotoxin properties [10], infection is not requested to induce deleterious effects in the intestinal epithelium [105] and nerve cells [106], through direct binding of the viral glycoprotein to cell surface glycosphingolipids, such as galactosylceramide [4,5].

## 6. Considerations on the Electrostatic Surface Potential

As discussed above, the surface electrostatic potential plays a major role in the selection mechanisms that are directly responsible for the emergence of new viral populations with increased tropism and/or infectivity. At this point, we must make an important distinction between kinetic effects and affinity enhancement [34]. Regarding the lipid raft recognition domains, it is clear that the increase in surface electrostatic potential does not translate into an increase in the affinity of viruses for gangliosides. There are several reasons for this. First, the interaction of viral glycoproteins with raft gangliosides involves several distinct gangliosides [62]. It is, therefore, possible to measure an overall avidity for this cluster of gangliosides, which is the sum of the affinity of each individual ganglioside for the viral glycoprotein. The variation in free energy (ΔG) associated with this multi-partner reaction is already very large, and it can no longer increase significantly [34]. Indeed, in the case of SARS-CoV-2, this ΔG shows very little variation from one variant to another, despite the accumulation of mutations in the spike protein. In contrast, the increase in surface potential gives a clear kinetic advantage, allowing the most electropositive viruses to adhere to target cells faster than their competitors.

This advantage is conferred by the particular associations of mutations concentrated in the V3 loop of HIV-1 gp120 [107] and at the surface of the NTD and of the RBD in the case of the spike protein of SARS-CoV-2 [34]. In the latter case, the NTD and the RBD can evolve in concert by increasing their surface electrostatic potential, which can be visualized when observing the upper face of the spike protein trimer. This is the case for all SARS-CoV-2 variants, from the original Wuhan strain to the Delta variant (Figure 3), but not for the Omicron series. This series of variants differs significantly from their predecessors because, for them, the increase in surface potential essentially concerns the RBD. This unexpected asymmetry explains why the T-index of the first Omicron variant is lower than that of the last Delta variant [72].

## 7. Impact on Immune Responses

The virus-cell recognition domains located at the top of gp120 and spike protein trimers are natural targets for anti-HIV and anti-coronavirus neutralizing antibodies. The V3 loop was initially characterized as the principal neutralization domain (PND) of HIV-1 [108]. Similarly, the top-exposed surfaces of the NTD and RBD contain neutralizing epitopes [73]. The natural evolution of viruses obviously concerns these neutralization zones, which explains the gradual loss of the effectiveness of the neutralizing antibodies over time. By taking, as references, the main neutralizing epitopes at the level of the NTD and the RBD, we have developed an immuno-neutralization index (IS index), which quantifies the resistance to neutralizing antibodies of any SARS-CoV-2 variant [73]. Very logically, we then demonstrated a perfect correlation between the value of the T-index and the IS index for the SARS-CoV-2 variants. The mutational profiles conferring the greatest increase in surface potential are those conferring the strongest resistance to the neutralizing antibodies directed against the Wuhan reference strain. In summary, the faster a virus is, the less it is neutralized. Conversely, slower viruses are more effectively neutralized. Overall, the evolution of viruses, therefore, favors the emergence of viruses that are increasingly rapid and less sensitive to neutralizing antibodies. This explains the loss of efficacy of the vaccine based on the Wuhan strain for the Delta variants [61] and, even more blatantly, for the variants of the Omicron series [109]. For HIV-1, the same process explains why the immune system of an infected individual systematically loses the race towards neutralization, while at the same time, increasingly rapid viruses with extended tropism emerge irremediably.

## 8. Key Role of Lipid Rafts

These selection mechanisms based on a single fundamental principle, the surface electrostatic potential, make it possible to understand multiple aspects of the structural dynamics of RNA viruses, illustrated here by HIV-1 and SARS-CoV-2. Given that the initial interaction of these viruses with the surface of host cells takes place at the level of the lipid rafts, it can be deduced that it is the molecular components of these rafts, mainly the gangliosides, that are at the origin of this dynamics of evolution. This has important consequences for the selection of protein receptors used by these viruses. First, these receptors must be associated with lipid rafts. Second, they must have an electronegative contact surface that can attract the electropositive surface glycoprotein domains of viruses. This is the case for the ACE2 receptor [110,111] and the CXCR4 co-receptor [102]. Thus, a similar mechanism of interaction with lipid rafts may apply for both HIV-1 and SARS-CoV-2.

The first step is the attraction of the trimeric spike by a lipid raft, based on virus-ganglioside interactions. To study these interactions, we developed a Langmuir balance system, allowing us to measure the surface pressure variations induced by HIV-1 gp120 on a calibrated glycosphingolipid monolayer at the air-water interface [112]. With this apparatus, we studied the interaction of several types of glycosphingolipids with various recombinant or purified gp120 coming from different HIV-1 isolates [13]. Subsequently, we used a miniaturized system developed by the Finnish company Kibron [113]. The advantage of the Langmuir monolayer system is that the experimenter can modulate the composition of the monolayer. For instance, lipid rafts can be mimicked by a combination of specific raft lipids (sphingomyelin, gangliosides, and cholesterol) in appropriate molecular ratios [114]. One can also prepare reconstituted hemimembranes corresponding to the cytoplasmic leaflet of the plasma membrane [115]. Since CD4 is lipid raft-associated protein, we built a mixed monolayer containing both raft lipids and the CD4 receptor [19]. To this end, we first prepared a monolayer of ganglioside GM3, and we added the CD4 protein on this monolayer. Then, we incubated this complex GM3-CD4 complex with HIV-1 gp120. The biphasic increase in surface pressure observed after the sequential addition of CD4 and gp120 on the GM3 monolayer indicated the formation of a trimolecular GM3-CD4-gp120 complex. From these experimental results, we have proposed a surfing model, according to which, the HIV-1 particle, docked on a GM3-CD4 raft, moves on the surface of the cell until it encounters a functional coreceptor (CCR5 or CXCR4) (Figure 7). This model also applies to SARS-CoV-2, but in this case, the ganglioside is GM1, the receptor is ACE2, and the co-receptor the TMPRSS2 protease. It emerges from the infection strategies of these two RNA viruses a common point, the lipid rafts, without which the infection cannot take place. A comparative analysis of the mechanisms of entry of HIV-1 and SARS-CoV-2 is summarized in Table 1. Both viruses share a preferential tropism for immune cells, mucosa, and neural cells. In addition, SARS-CoV-2 may also enter the body through the infection of ocular cells [116].

## 9. Common Mechanisms of Pathology

The similarities between HIV-1 and SARS-CoV-2 do not concern only the molecular mechanisms involving a controlled succession of protein-raft interactions. There are also interesting commonalities in the pathological consequences of infection with these viruses (Table 2). First, the site of infection is predominantly mucosal. It is the respiratory epithelium in the case of SARS-CoV-2 [117] and the vaginal or rectal mucosa for HIV-1 [118,119,120,121]. Secondly, these two viruses cause severe gastrointestinal [122,123,124] and neurological pathologies [125,126]. In these two cases, the infection is not mandatory because the gp120 of HIV-1, just like the spike protein of SARS-CoV-2, has the capacity to act as a toxin. Thus, in 1995, we identified HIV-1 gp120 as a virotoxin capable of triggering a signal transduction cascade after stimulation of intestinal epithelial cells. After binding its glycosphingolipid receptor, galactosylceramide (GalCer) [4], gp120 induces a release of intracellular calcium [7], which, in turn, causes the depolymerization of microtubules [8] and cell dedifferentiation, hypersecretion, and malabsorption [6,10]. In collaboration with the team of F. Clayton, we have identified the GPR15 co-receptor as being the armed component of this virotoxin-induced HIV-1 enteropathy [105]. Interestingly, COVID-19 is a disease that can also present complications at the intestinal level [127,128], potentially linked to the virotoxin properties of the spike protein [129,130,131,132,133]. Similarly, both RNA viruses may induce neurological symptoms, consistent with the demonstration that both HIV-1 gp120 and the SARS-CoV-2 spike protein can perturb and penetrate in the brain via the blood–brain barrier [134,135,136,137,138]. Taken together, all these observations show that this retrovirus and this coronavirus share common molecular mechanisms at the level of their entry mechanisms, a potent virotoxin activity of their receptor-binding protein and, thus, the ability to induce pathological symptoms on intestinal and brain tissues. It is also interesting to note that the SARS-CoV-2 infection can induce an immunosuppression [139,140], a hallmark of HIV-1 disease [141].

Finally, a striking observation concerning both viruses came from the recent Nobel Prize for Svante Pääbo. The main genetic risk factor for severe forms of COVID-19 is a fragment of chromosome 3 inherited from Neanderthals [142]. This portion of DNA regulates the expression of several chemokine receptors, including the HIV-1 co-receptor CCR5. In carriers of the COVID-19 risk haplotype, CCR5 is down-regulated. These individuals then have a markedly reduced risk of HIV-1 infection [143]. The evolution of viruses, as well as virus-host co-evolution, brings us many surprises.

## 10. Therapeutic Applications

How can the knowledge gained from decades of HIV-1 studies be applied to SARS-CoV-2 and future pandemics? First and foremost, we need to incorporate the fact that the vast majority of viral infections require the functional integrity of lipid rafts [144,145,146,147]. We must consider viruses as lipid raft hijackers [148,149] and focus our therapeutic strategies accordingly [15,150,151]. However, this attack style has not been exploited as it deserves, probably because of the propensity of biologists to generally consider membrane proteins outside of their lipid context. This is a big mistake, since raft glycosphingolipids control several key pathological mechanisms associated with RNA virus infection. For instance, one hallmark of patients at an early stage of HIV-1 infection is a dysregulation of glycosphingolipid metabolism in peripheral blood mononuclear cells [152]. There is an accumulation of Gb3 and GM3 associated with the appearance of anti-Gb3 and anti-GM3 antibodies that may contribute to immune suppression [15,152].

In vitro, it suffices to temporarily disorganize the lipid rafts by reversible treatment with methyl-β-cyclodextrin (which extracts cholesterol from the membranes) or PPMP (a metabolic inhibitor of glycosphingolipid biosynthesis) to prevent these viruses to infect the cells [153]. Of course, it will be necessary to be more imaginative to design safe and effective drugs compatible with the treatment of humans, but the task is not insurmountable. This is also the challenge we set ourselves at the turn of the 2000s, when we transferred our knowledge acquired on retroviruses to the field of molecular neuroscience.

Our first discovery at the virology–neurology frontier was to demonstrate a structural homology between the V3 loop of HIV-1 and the membrane-induced folding of amyloid proteins responsible for Alzheimer’s, Creutzfeldt–Jakob, and Parkinson’s diseases [25]. Then, we cracked the biological code controlling the interaction of these proteins with brain gangliosides [26]. This strategy allowed us to design the therapeutic peptide AmyP53, which is the first molecule voluntarily created to bind to the gangliosides recognized by amyloid proteins [154]. As expected, the AmyP53 peptide prevents these proteins from binding to the lipid rafts of brain cells, which neutralizes their neurotoxic potential [155,156]. It is, therefore, possible to develop therapeutic molecules acting at the level of membrane rafts [157,158]. It is, nevertheless, very important to consider rafts as structures with emergent properties, which can vary according to the molecular assemblies of their components. The periphery of a raft is not equivalent to its internal areas [62]. The presence of particular proteins, which can, themselves, interact with the gangliosides and the cholesterol of the rafts, is also a parameter to consider [159]. In this respect, it is important to note that alternative receptors, such as CD147, neuropilin, or CD26 (Table 1), which may facilitate SARS-CoV-2 entry into cells expressing low ACE2 levels [160], are also associated with lipid rafts [161,162,163]. The same rule applies for the main alternative receptors used by HIV-1: the membrane protein DC-sign [164] and the glycosphingolipid galactosyl ceramide, all functionally associated with lipid rafts [17,165,166].

A clear advantage of targeting rafts, rather than viral proteins, is that gangliosides have a stable biochemical structure, while the viral proteins that recognize them evolve. The prevention or treatment strategies based on these proteins are, therefore, doomed to failure when faced with RNA viruses that have a high mutational potential. In support of these considerations, we can only note the absence of an anti-HIV vaccine, despite decades of research efforts and the progressive loss of effectiveness of anti-SARS-CoV-2 vaccines based on the sequence of the original virus spike protein.

In the beginning of the COVID-19 pandemic, long before vaccines were available to populations, a high interest was given to testing repositioned drugs [167]. This is how hydroxychloroquine and azithromycin, alone or in combination, were identified as potential solutions [168,169]. According to our global model of viral infections, it was logical to test the ability of these molecules to bind to lipid rafts. Using a dedicated molecular modeling approach, we showed that hydroxychloroquine displays an excellent affinity for GM1 gangliosides clustered in a lipid raft environment [29]. Using the same approach, we showed that azithromycin can interact with the ganglioside-binding domain of the NTD of the SARS-CoV-2 protein [30]. Overall, our data explain the synergistic effect of hydroxychloroquine and azithromycin to prevent SARS-CoV-2 infection in vitro [28]. We anticipate that targeting lipid rafts (with either repositioned or specifically designed drugs) is certainly a strategy for the future, in the case we are confronted with new virus pandemics [81]. As a broad-spectrum approach that dismisses the “one virus-one drug” rule [170], this strategy shatters the old classifications of therapeutic agents, such as antibiotics or antiparasitics, whose therapeutic action is generally considered specific to these organisms. The demonstration of the antiviral effects of azithromycin [30,171] has taught us not to take such rigid positions.

Academic research has already produced several interesting lipid raft-inspired drugs, such as glycosphingolipid analogues [149,172,173,174,175,176,177,178,179] or synthetic peptide-targeting gangliosides [180,181,182]. Since the COVID-19 pandemic caused by SARS-CoV-2, several alerts for potentially dangerous viruses for humans have been issued by academic researchers and/or the WHO. The two most recent events are the outbreak of Monkeypox virus [81] and the demonstration that simian hemorrhagic fever virus (SHFV) can infect human cells [183]. As expected, both viruses use lipid rafts as a portal of entry into human cells [81,184]. Faced with these dangers, it is urgent to put in place a reasoned health monitoring strategy and to take advantage of the knowledge acquired on the biology of HIV, still one of the most studied viruses in the history of biology: 404,000 scientific articles for “HIV” vs. 184,000 for “SARS-CoV-2” from a total 1,379,000 entries for “virus”, according to Pubmed (https://pubmed.ncbi.nlm.nih.gov/, accessed 15 November 2022). In this context, lipid raft-based therapies will undoubtedly be at the forefront of the fight.

## Figures and Tables

**Table 1:** Comparative analysis HIV-1 and SARS-CoV-2 entry mechanisms.

Characteristic	HIV-1	SARS-CoV-2
**Genome**	RNA 10 kb	RNA 30 kb
**Entry into host cells** **and propagation**	Lipid raft mediated fusion or endocytosis, cell-cell fusion	Lipid raft mediated fusion or endocytosis, cell-cell fusion
**Main receptor**	CD4	ACE2
**Alternative receptor**	DC-sign, galactosylceramide	CD147, neuropilin, CD26
**Receptor binding protein**	Gp120	Spike protein S1
**Fusion domain**	Gp41	Spike protein S2
**Raft cofactor**	GM3, Gb3, cholesterol	GM1, cholesterol
**Raft binding domain**	V3 loop	Spike protein NTD
**Coreceptor**	CCR5, CXCR4, GPR15	TMPRSS2
**Mucosal site of infection**	Vaginal or rectal mucosa	Respiratory mucosa
**Target cells**	Lymphocytes Macrophages Intestinal cells Neural cells	Respiratory epithelial cells Intestinal epithelial cells Lymphocytes Macrophages Neural cells Ocular cells
**Virotoxin**	Gp120	Spike protein

**Table 2:** Similarities between HIV-1 and SARS-CoV-2 pathogenesis.

Characteristic	HIV-1	SARS-CoV-2
**Animal reservoir**	Non-human primates	Bats, minks, hamsters, domestic animals, zoo animals (non-human primates), wild animals
**Low fidelity enzyme**	Reverse transcriptase (RT)	RNA polymerase (RdRp)
**Genetic variability**	Quasi-species, subtypes	Quasi-species, variants, recombinants
**Mutational pattern**	Mutations, insertions, deletions, recombinants	Mutations, insertions, deletions, recombinants
**Selection pressure**	Antiretroviral drugs, immunity	Immunity, vaccines
**Pathology**	lymphopenia Gastrointestinal dysfunction Microbiome alteration Neurological disorders Thrombosis Proinflammatory cytokines synthesis	lymphopenia Gastrointestinal dysfunction Microbiome alteration Neurological disorders Thrombosis Proinflammatory cytokines synthesis
